# Endoscopic Transnasal Excision of Foramen Ovale Schwannoma: A Case Report and Literature Review

**DOI:** 10.1155/crot/1152945

**Published:** 2025-08-08

**Authors:** Hamdan Ahmed Pasha, Fatima Syed Amanullah, Muhammad Shahzaib Arshad, Isra Ahmed, Noor Amanullah, Ainulakbar Mughal

**Affiliations:** ^1^Department of Surgery, The Aga Khan University, Karachi 74800, Pakistan; ^2^Department of Undergraduate Education, University of Toronto, Mississauga, Canada

**Keywords:** endoscopic transnasal approach, foramen ovale, minimally invasive surgery, trigeminal schwannoma

## Abstract

**Background:** Schwannomas are benign, slow-growing tumors that develop from Schwann cells in the nerve sheath and can occur in peripheral, cranial, or autonomic nerves. Foramen ovale schwannomas are a rare variant in the head and neck region representing 1%-2% of all intracranial schwannomas, with parapharyngeal space involvement complicating surgical resection.

**Case Presentation:** A 48-year-old male presented with headaches after an episode of light-headedness along with facial pain. MRI showed a well-defined lesion measuring 50 × 40 × 20 mm in the left masticator space, extending superiorly up to the left temporal lobe. CT imaging revealed a 28 × 25 × 31-mm lesion in the left masticator space, extending through the foramen ovale into the medial left temporal lobe. The tumor was classified as Type ME under the Yoshida and Kawase system, involving both the middle cranial fossa and extracranial extension.

**Management:** A minimally invasive endoscopic transnasal approach was used for tumor resection, prioritizing preservation of the internal maxillary and carotid arteries. This approach was selected for its reduced morbidity and quicker postoperative recovery, as the tumor's medial location made it amenable to endoscopic access. The patient had an uneventful recovery. No new neurological deficits were reported at follow-up, and facial pain improved significantly.

**Conclusion:** This report reviews the current literature on the diagnosis and management of trigeminal schwannoma, highlighting minimally invasive techniques as effective alternatives to traditional surgical approaches.

## 1. Introduction

Schwannomas are benign tumors that develop from Schwann cells in the nerve sheaths and can occur in peripheral, cranial, or autonomic nerves. Trigeminal schwannomas, which represent 1%-2% of all intracranial schwannomas, are uncommon and typically arise in individuals between the 40 and 60 years, with a slightly higher incidence in females [1]. These tumors tend to present with facial pain, numbness, or a burning sensation [2]. Both CT and MRI play a critical role in the diagnosis with the former allowing for better visualization of the skull base anatomy and the latter aiding in definitive diagnosis [3]. Extracranial spread of trigeminal schwannomas in the masticator or parapharyngeal space makes these tumors difficult to surgically resect. This report focuses on a rare case of trigeminal schwannoma with extracranial extension through the foramen ovale into the parapharyngeal space, successfully resected using a minimally invasive transnasal approach.

## 2. Case Presentation

### 2.1. Patient History and Examination

A 48-year-old male with a history of hypertension presented to the otolaryngology clinic with a 2-3-month history of worsening headaches and facial pain. The symptoms had an insidious onset and progressively worsened over time. The patient described his headaches as sharp, electric-like spasms lasting 2–5 min and reported them to be severely painful. On examination, the patient exhibited an absent corneal reflex and sixth nerve palsy. Initially, he was managed with analgesics for his headache and later an MRI of the head was performed due to ongoing symptoms. Testing for neurofibromatosis Type 2 (NF2) was not performed due to the unavailability of genetic testing at our center.

### 2.2. Radiological Findings

**Figure 1:**
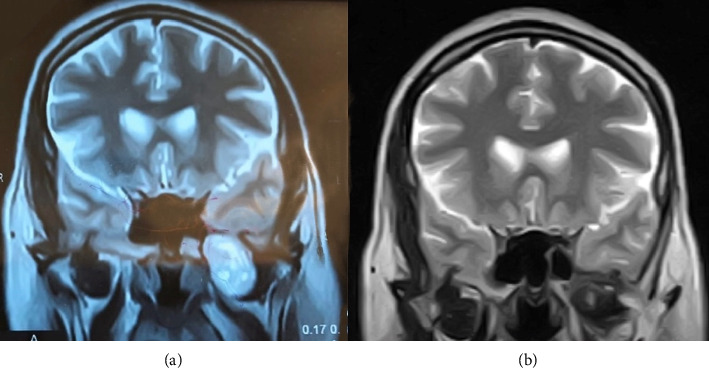
(a)Preoperative MRI shows a lesion measuring 50 × 40 × 20 mm in the left masticator space, extending superiorly up to the left temporal lobe. (b) Postoperative MRI done at 3 month interval showing complete removal of the tumor.

**Figure 2:**
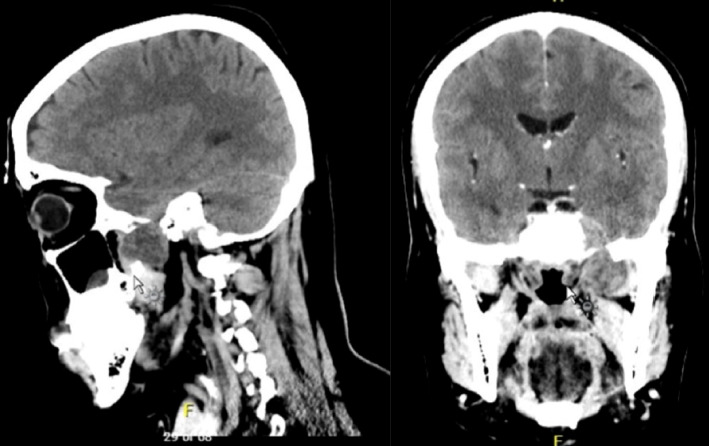
Sagittal and coronal CT views showing trigeminal schwannoma with intracranial extension.

MRI showed a well-defined lesion measuring 50 × 40 × 20 mm in the left masticator space, extending superiorly up to the left temporal lobe (Figure 1). The lesion was causing displacement of the adjacent brain parenchyma. CT revealed a well-defined lesion with patchy enhancement in the left masticator space, superior to the left lateral pterygoid muscle. The lesion abutted the lateral pterygoid plate and the cavernous segment of the internal carotid artery, with no evidence of thrombosis. Intracranial extension was observed through the foramen ovale into the medial part of the left temporal lobe. The lesion measured approximately 28 × 25 × 31 mm in anteroposterior, transverse, and craniocaudal dimensions (Figure 2). No bony erosion was evident, and there was no extension into the sphenoid sinus or orbit.

The brain parenchyma appeared normal, with intact gray and white matter differentiation. There was no evidence of intracranial hemorrhage, established infarction, or edema. Midline structures were preserved, with no evidence of midline shift or hydrocephalus. Ventricular and extra-axial CSF spaces were unremarkable, and the posterior fossa structures showed no abnormalities.

Additional findings included a retention cyst in the left maxillary sinus. The remaining paranasal sinuses and mastoid air cells appeared normal.

### 2.3. Surgical Approach

**Figure 3:**
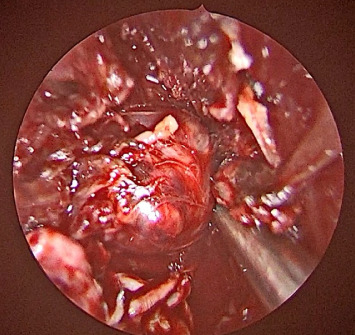
Intraoperative view of the encapsulated tumor exposed after removal of the posterior maxillary wall and separation of neurovascular bundles.

The tumor was in the left masticator space, posterior to the pterygoid muscles, and extended up to the foramen ovale. A minimally invasive transnasal endoscopic approach was used to preserve adjacent critical structures. First, the left maxillary sinus ostium and the sphenoid sinus ostium were widened to enhance visualization and access to deeper anatomical regions. A left endoscopic medial maxillectomy was then performed. The posterior wall of the maxillary sinus was resected to expose the underlying pterygoid muscles. The pterygoid muscles were then excised to provide direct access to the tumor (Figure 3). The internal maxillary artery was identified and ligated followed by careful separation of the carotid artery. The tumor was then successfully delineated and prepared for excision.

H&E staining shows alternating Antoni A and Antoni B areas. Antoni A areas display densely packed spindle cells with palisading nuclei, while Antoni B regions are hypocellular and loosely arranged. Verocay bodies (black arrow) are evident as rows of aligned Schwann cell nuclei. Immunohistochemistry shows strong S-100 positivity, confirming Schwann cell origin. No features of malignancy such as atypical mitoses or necrosis were observed.

### 2.4. Postoperative Course and Follow-Up

**Figure 4:**
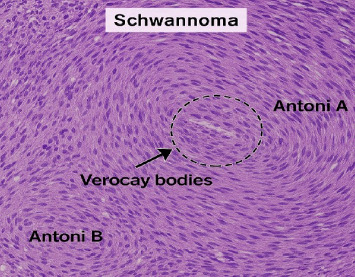
Histopathological features of trigeminal schwannoma.

The patient had an uneventful postoperative recovery and was discharged after 2 days. Follow-up at 7- and 14 days postsurgery revealed no signs of recurrence, and the patient reported improvement in his condition. Importantly, no new or persistent neurological deficits were observed on clinical evaluation. Histopathological examination confirmed the diagnosis of schwannoma, revealing characteristic Antoni A and B areas with verocay bodies and strong S-100 positivity (Figure 4).

## 3. Discussion

Trigeminal schwannomas typically manifest between the 20 and 30 years and are characterized by trigeminal nerve dysfunction [4, 5]. In their early stages, these tumors are often asymptomatic, but as they increase in size, common clinical features begin to emerge, such as abnormalities in the corneal reflex and facial paresthesia, affecting one or more branches of the trigeminal nerve [5]. With continued growth, patients may develop additional symptoms, including auditory impairment, muscle incoordination, ocular dysfunction, and elevated intracranial pressure [6]. Facial pain is another frequent symptom, though it is essential to distinguish this from trigeminal neuralgia to avoid misdiagnosis. Trigeminal neuralgia typically manifests as brief, stabbing pain, whereas the pain associated with trigeminal schwannomas is more often chronic, burning, and accompanied by numbness [4–6]. The tumor's location and growth pattern play a significant role in determining the clinical presentation, as compression of adjacent structures can lead to specific dysfunctions. For instance, involvement of the cavernous sinus may result in exophthalmos and vision loss and compression of the vestibulocochlear nerve or blockage of the eustachian tube can cause hearing loss [4].

Trigeminal schwannomas can arise from any part of the trigeminal nerve, extending from its root in the posterior cranial fossa to the peripheral extracranial branches. Jefferson initially classified these tumors into three types: Type A, originating from Gasser's ganglion in the middle cranial fossa; Type B, from the trigeminal nerve root in the posterior cranial fossa; and Type C, characterized by hourglass-shaped tumors involving both cranial fossae. Type D was later added for extracranial growths [7]. Yoshida and Kawase expanded this into a six-type system, now widely used, including Type P (isolated posterior cranial fossa lesions from the nerve root), Type M (middle cranial fossa tumors from Gasser's ganglion or cavernous sinus), and Type E (extracranial tumors, with E1 involving orbital extension and E2 affecting the pterygopalatine or infratemporal fossa). Combinations of these types—MP, ME, and MPE—are also widely recognized [8]. Trigeminal schwannomas can be further classified by size: small (< 2 cm), medium (2-3 cm), and large (> 3 cm), with some classifications designating tumors larger than 4 cm as giant schwannomas [9].

Due to their complex anatomical involvement and vague clinical symptoms, trigeminal schwannomas can be difficult to diagnose. MRI plays a critical role in their identification, typically revealing isointense or slightly hyperintense signals on T1-weighted images and hyperintense signals on T2-weighted images, with pronounced enhancement after contrast administration [2, 9]. Thin T2-weighted CISS 3D axial sequences are particularly useful for evaluating the cisternal segment of the trigeminal nerve, especially in cases involving the skull base [1, 10]. CT scans are supportive, particularly for skull base tumors, which may appear as uniformly enhancing masses with accompanying bone remodeling [10]. However, histopathological confirmation is necessary for definitive diagnosis, as MRI alone cannot reliably distinguish trigeminal schwannomas from other encapsulated benign cranial nerve tumors [2].

Management of trigeminal schwannomas requires a multimodal assessment, with the primary objectives being the minimization of neurological symptoms, maintenance of cranial nerve function, and control of mass effect [11]. The available treatment options are broadly categorized into surgery, radiosurgery, and radiotherapy [7]. Surgical approaches are often determined by the tumor's anatomy and size [12]. Trigeminal schwannomas classified as Type ME are usually resected through the orbitozygomatic infratemporal approach as outlined by Yoshida and Kawase [8]. However, in our case, the transnasal endoscopic approach was chosen due to its minimally invasive nature, reduced operative morbidity, and suitability for the lesion's medial location. Advances in surgical techniques have focused on achieving higher resection rates while reducing recurrence [5]. Microsurgery has significantly advanced treatment in this area, with neuroendoscopy also becoming a viable option for managing different types of trigeminal schwannomas [13].

For the preservation of facial sensation, minimally invasive procedures such as radiosurgery have become an acceptable alternative, with its subtypes including gamma knife radiosurgery (GKS) and linear accelerator-based radiosurgery (LINAC), with GKS being the more commonly used technique [5, 14, 15]. Careful consideration is needed when selecting this modality, as unwanted tumor expansion has been reported in studies related to increased margin doses. In cases where tumors compress the brainstem, surgery may be preferred over GKS [16]. Due to the common late presentation of trigeminal schwannomas, when mass effects are already significant, surgical resection typically remains the first-line treatment. However, radiotherapy can still be considered as a postoperative option for local control [17].

## 4. Conclusion

The endoscopic nasal approach has emerged as an effective method for resection, offering minimal tissue disruption and enhanced intraoperative visualization. We encourage further reporting of trigeminal schwannoma cases and their management to build evidence supporting this minimally invasive technique as a reliable treatment option.

## Data Availability

All relevant data related to this case report are included within the manuscript.
